# Spatio-temporal analysis of the double burden of malnutrition in children from the Brazilian semi-arid region

**DOI:** 10.11606/s1518-8787.2025059006605

**Published:** 2025-12-08

**Authors:** Maisa Póvoa de Oliveira, Cíntia Pereira Donateli, Daniela Mayumi Usuda Prado Rocha, Helen Hermana Miranda Hermsdorff

**Affiliations:** I Universidade Federal de Viçosa. Departamento de Nutrição e Saúde. Viçosa, MG, Brasil; II Universidade Federal de Viçosa. Instituto de Políticas Públicas e Desenvolvimento Sustentável. Viçosa, MG, Brasil

**Keywords:** Nutritional Status, Child Nutrition, Primary Health Care, Secondary Data Analysis, Prevalence, Geographic Mapping

## Abstract

**OBJECTIVE:**

To analyze the temporal trend of the double burden of malnutrition in the Brazilian semi-arid and non-semi-arid regions, and to assess its spatial distribution in the Brazilian semi-arid region among children aged 5 to 9 years, between 2008 and 2022.

**METHODS:**

Ecological time-series study based on public reports, using macrodata of body mass index-for-age and height-for-age from the *Sistema de Vigilância Alimentar e Nutricional* (SISVAN – Brazilian Food and Nutritional Surveillance System). For temporal analysis of the prevalence of wasting, overweight, stunting, undernutrition, and the double burden of malnutrition in the semi-arid and non-semi-arid regions, Prais-Winsten regression models were used to estimate the annual percentage change (APC%) between 2008 and 2022. To evaluate spatial distribution in the Brazilian semi-arid region, maps showing the temporal evolution of the prevalence of overweight, undernutrition, and the double burden of malnutrition were constructed.

**RESULTS:**

Between 2008 and 2022, 48.7 million children aged 5 to 9 years were registered in SISVAN-Web for body mass index-for-age and height-for-age indices. Approximately 28.1% of these lived in the Brazilian semi-arid region. The prevalence of the double burden of malnutrition in the semi-arid region exceeded 20%, with an increasing trend among children aged 7 to 9 years (APC = 0.61%; p < 0.05). Overweight showed a rising trend, while undernutrition decreased across all strata except among the Indigenous population, which remained stable (APC = −1.12%; p > 0.05). APCs in the semi-arid region were higher than in the non-semi-arid region.

**CONCLUSION:**

The study highlights a high prevalence of the double burden of malnutrition in the Brazilian semi-arid region, especially among children aged 7 to 9 years. Therefore, it is crucial to promote health actions and malnutrition prevention, strengthening existing policies and prioritizing the most vulnerable populations.

## INTRODUCTION

In populations facing socioeconomic vulnerability, the prevalence of overweight has increased rapidly, while the decline in undernutrition has occurred slowly^1^. This phenomenon results in the presence of the double burden of malnutrition (DBM), defined as the coexistence of overweight and undernutrition in populations, families, or individuals^1^.

The presence of this dual burden of malnutrition constitutes an essential public health issue^4^. Globally, it is estimated that in 2022, 190 million children and adolescents aged 5 to 19 years were underweight, while 160 million were overweight, indicating a global DBM prevalence of 17.8%^4^. In Brazil, the situation is also concerning, with an estimated 6.4 million children overweight and 3.1 million having progressed to obesity^5^. At the same time, the prevalence of undernutrition, although having declined significantly in recent decades, showed an increase from 2018, reaching 5.3% in 2021 among children and adolescents aged 0 to 19 years^6^.

Childhood overweight, in addition to being a risk factor for the early development of other non-communicable chronic diseases (NCDs)—such as type 2 diabetes, cardiovascular disease, and cancer—is associated with reduced mobility, joint pain, and psychological disorders influenced by social stigma and bullying, including depression and eating disorders^7,8^. Chronic undernutrition, in turn, can cause short- and medium-term damage, such as delays in growth and cognitive development, affecting motor and learning skills; weakening the immune system; and long-term consequences such as predisposition to NCDs—similar to overweight—and, in severe cases, may lead to death^9^. Therefore, the presence of DBM in childhood can have repercussions for the entire population, both in terms of health and socioeconomic development, since it affects school performance, reduces productivity and human capital, increases morbidity and mortality, and diminishes quality of life^10,11^.

In the context of DBM, assessing and monitoring the nutritional status of children —particularly in regions of higher vulnerability, such as the Brazilian semi-arid region (SAR)—becomes an essential tool for identifying epidemiological patterns, social determinants, and both early and future nutritional issues. The SAR, the most populous semi-arid region in the world with approximately 31 million people^12^, is characterized by high solar radiation and great variability in rainfall, with long periods of drought that influence food production and distribution^13,14^. In addition to climatic vulnerability, the region faces socioeconomic vulnerabilities, including limited income, education, and sanitation^15^.

Moreover, the literature lacks information on the nutritional status of children in the SAR, as longitudinal data are limited. This absence of evidence hinders a comprehensive understanding of the specific challenges faced by children in this region, which has unique socioeconomic, environmental, and climatic characteristics. Knowing and monitoring the nutritional status of the SAR’s child population, as well as how it compares to other regions, is crucial for setting priorities. In Brazil, this monitoring is conducted in Primary Health Care (PHC) services via anthropometric measurements recorded in the *Sistema de Vigilância Alimentar e Nutricional* (SISVAN – Brazilian Food and Nutritional Surveillance System)^16^. For children aged 5 to 9 years, reports are available with information on weight-for-age, height-for-age (H/A), and body mass index-for-age (BMI/A) indices^16^.

Given the need for research describing the DBM situation among children in the SAR, this study aims to analyze the temporal trend of DBM in the SAR and in the non-semi-arid region of Brazil (NSAR) and to assess its spatial distribution in the SAR among children aged 5 to 9 years, from 2008 to 2022.

## METHODS

### Study Design and Data Source

This was an ecological time-series study based on secondary data available in the SISVAN, aggregated by municipality. The study aims to assess the prevalence of the double burden of malnutrition (DBM), defined as the coexistence of undernutrition and overweight, among children aged 5 to 9 years residing in the Brazilian SAR from 2008 to 2022.

SISVAN provides data on food and nutrition surveillance activities for individuals served in PHC. Records are entered by professionals from basic health units during activities conducted in the e-SUS Primary Care system and the Bolsa Família Program Management System. These data are periodically and automatically transferred to the SISVAN platform^16,17^.

Thus, anthropometric data on children’s nutritional status for the period from 2008 to 2022, aggregated by municipality, were extracted from publicly accessible consolidated reports available for download on the SISVAN-Web platform^a^.

### Geographic Characterization

**Figure 1:**
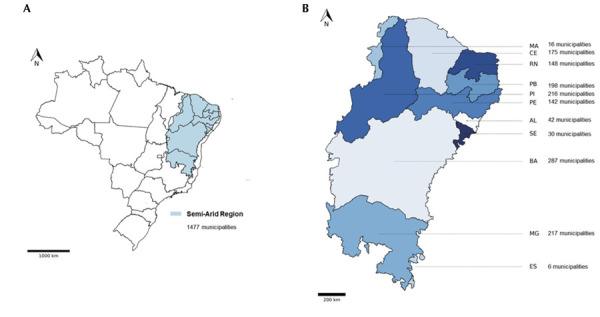
Demarcation of the semi-arid region within the Brazilian territory (A) and demarcation of the federative units that make up the Brazilian semi-arid region (B).

MA: Maranhão; CE: Ceará; RN: Rio Grande do Norte; PB: Paraíba; PI: Piauí; PE: Pernambuco; AL: Alagoas; SE: Sergipe; BA: Bahia; MG: Minas Gerais; ES: Espírito Santo.

Source: CONDEL/SUDENE, 2021^18^.

The units of analysis in this study were the municipalities of Brazil, grouped into the SAR and non-semi-arid region (NSAR). The SAR was defined according to Resolution No. 150 of the Deliberative Council of the Northeast Development Superintendence, dated December 13, 2021, which establishes that, among Brazil’s 5,570 municipalities, 1,477 belong to the SAR. This region encompasses 11 Brazilian states: Maranhão, Piauí, Ceará, Rio Grande do Norte, Paraíba, Pernambuco, Alagoas, Sergipe, Bahia, Minas Gerais, and Espírito Santo^18^ (Figure 1). The remaining 4,093 municipalities were classified as part of the NSAR.

### Participants

The study included all children aged 5 to 9 years, of both sexes, with recorded anthropometric measurements in SISVAN for each municipality-year from 2008 to 2022. These data were then grouped for analysis comparing the SAR and NSAR.

### Data Analysis

Nutritional status was assessed according to the World Health Organization (WHO) Growth Reference (2007). Based on the BMI-for-age (BMI/A) index^12^, children were classified as overweight (> z-score +1 and ≤ +2), obese (> z-score +2 and ≤ +3), or severely obese (> z-score +3). Thinness was defined as ≥ z-score −3 and < −2, and severe thinness as < z-score −3, also according to BMI/A^16^. For the height-for-age (H/A) index, children were classified as having very short stature (z-score < −3) or short stature (≥ z-score −3 and < −2)^16^. From these classifications, the prevalence of the anthropometric outcomes of interest was estimated as follows.

The prevalence of wasting was obtained by grouping the categories of thinness and severe thinness, using the formula:


$$\text{ Prevalence of wasting }(\%)=\frac{\text{ thinness }(n)+\text{ severe thinness }(n)}{\text{ total assessed by BMI/A }(n)}\times 100$$

The prevalence of overweight was estimated by grouping the categories of overweight, obesity, and severe obesity^19^, as follows:


$$\mathrm{Prevalence}\mathrm{of}\mathrm{overweight}(\%)=\frac{\mathrm{overweight}(n)+\mathrm{obesity}(n)+\mathrm{severe}\text{ obesity }(n)}{\mathrm{total}\text{ assessed by BMI/A }}\times 100$$

The prevalence of stunting was obtained by grouping the classifications of very short and short stature for age:


$$\text{ Prevalence of stunting }(\%)=\frac{\begin{array}{c} \text{ very short stature }(n)+\\ \text{ short stature }(n) \end{array}}{\text{ total assessed by }H/A(n)}\times 100$$

The prevalence of undernutrition was defined as the presence of acute (wasting) or chronic (stunting) undernutrition^16,19^, according to the formula:


$$\text{ Prevalence of undernutrition }(\%)=\frac{\mathrm{wasting}(n)+\mathrm{stunting}(n)}{\begin{array}{c} \text{ total assessed by BMI/A }(n)+\\ \text{ total assessed by }H/A(n) \end{array}}\times 100$$

The prevalence of the double burden of malnutrition (DBM)—the focus of this study —was defined as the simultaneous presence of undernutrition (wasting and stunting) and overweight (overweight, obesity, and severe obesity)^1,10,19^, using the formula:


$$\text{ Prevalence of DBM }(\%)=\frac{\text{ overweight }(n)+\text{ undernutrition }(n)}{\text{ total assessed by BMI/A }(n)+}\times 100$$

The temporal trend of nutritional status prevalence—according to the territorial divisions of the SAR, NSAR, and Brazil as a whole for each year—was analyzed using Prais-Winsten regression models, a method recommended for ecological studies to control for autocorrelation of residuals between years^20^. Data were further stratified by sex, race/skin color, and age group for the SAR. The annual percentage change (APC) and corresponding 95% confidence intervals (95%CI) were estimated using the formula: (−1 + 10^β) × 100, in which β represents the regression coefficient from the Prais-Winsten model. Non-significant p-values (p ≥ 0.05) indicated a stable trend, whereas significant values (p < 0.05) showed an increasing or decreasing trend, depending on the sign of the variation^20^. All data analyses were performed using R software, version 4.3.3.

Maps representing the delimitation of the SAR^18^ were generated using the GeoPandas and Matplotlib libraries in Python, version 3.11.5, based on cartographic shapefiles corresponding to the boundaries of municipalities and states from the *Instituto Brasileiro de Geografia e Estatística* (Brazilian Institute of Geography and Statistics)^21^. Additional maps were produced to illustrate the temporal evolution of the spatial distribution of overweight, undernutrition, and DBM prevalence in the SAR for the years 2008, 2015, and 2022

### Ethical Aspects

The study employed secondary public-domain data and was therefore exempt from review by an institutional research ethics committee for studies involving human subjects.

## RESULTS

In Brazil, from 2008 to 2022, a total of 48,714,371 records of children aged 5 to 9 years were identified in SISVAN-Web for the BMI-for-age (BMI/A) index, and 48,712,533 records for the height-for-age (H/A) index. Of these, 13,704,166 (28.13%) and 13,701,835 (28.13%) were from children residing in the semi-arid region (SAR), respectively.

The prevalence of wasting, stunting, and undernutrition showed decreasing trends in both regions analyzed. In contrast, the prevalence of overweight exhibited an increasing trend across all regions, with the highest percentage variation observed in the SAR (APC = 2.20%; 95%CI: 1.79 to 2.62). The prevalence of the double burden of malnutrition (DBM) remained stable in both the SAR and non-semi-arid region (NSAR) (Table 1).

**Table 1:** Temporal trend of the prevalence of the double burden of malnutrition among children aged 5 to 9 years registered in the SISVAN, by region, Brazil, 2008–2022.

Parameter	Period															APC (%)	95%CI	Trend
	2008	2009	2010	2011	2012	2013	2014	2015	2016	2017	2018	2019	2020	2021	2022			
Annual prevalence of wasting (%)																		
SAR	8.05	8.25	8.34	7.76	7.52	7.41	6.84	6.36	6.67	6.33	5.68	6.48	6.43	6.37	6.33	−2.12	−3.08 to −1.15	Decreasing
NSAR	6.37	6.36	6.50	6.22	5.93	6.04	5.77	5.10	5.19	5.07	4.68	5.41	5.26	5.08	4.96	−2.02	−2.82 to −1.21	Decreasing
Brazil	6.92	6.95	7.06	6.69	6.40	6.45	6.09	5.46	5.60	5.43	4.97	5.70	5.56	5.41	5.28	−2.20	−3.01 to −1.38	Decreasing
Annual prevalence of overweight (%)																		
SAR	23.41	23.88	24.67	26.15	26.19	26.58	28.03	28.60	29.18	28.99	29.43	28.05	31.34	33.41	31.36	2.20	−1.79 to −2.62	Increasing
NSAR	24.51	25.16	25.45	26.56	26.95	27.37	27.60	28.79	29.22	28.80	29.27	28.18	31.91	34.15	31.23	1.91	−1.51 to −2.32	Increasing
Brazil	24.15	24.76	25.21	26.44	26.72	27.13	27.73	28.74	29.21	28.86	29.31	28.15	31.76	33.96	31.26	2.01	−1.61 to −2.40	Increasing
Annual prevalence of stunting (%)																		
SAR	14.34	13.90	12.84	12.64	11.16	10.18	9.85	8.98	9.66	9.11	8.58	7.75	8.82	8.65	7.71	4.25	−5.33 to −3.15	Decreasing
NSAR	12.81	12.55	11.79	11.29	9.86	9.89	9.90	8.61	8.90	8.63	8.33	7.72	8.79	8.23	6.77	3.85	−4.63 to −3.06	Decreasing
Brazil	13.31	12.97	12.11	11.69	10.25	9,98	9.89	8.72	9.11	8.76	8.40	7.73	8.80	8.34	6.99	4.03	−4.86 to −3.20	Decreasing
Annual prevalence of undernutrition (wasting + stunting) (%)																		
SAR	11.19	11.08	10.59	10.20	9.34	8.79	8.35	7.67	8.16	7.72	7.13	7.11	7.63	7.11	7.02	3.36	−4.40 to −2.30	Decreasing
NSAR	9.59	9.45	9.14	8.75	7.89	7.97	7.84	6.85	7.05	6.85	6.50	6.57	7.03	6.65	5.87	−3.21	−3.95 to −2.47	Decreasing
Brazil	10.12	9.96	9.58	9.19	8.32	8.22	7.99	7.09	7.36	7.09	6.68	6.71	7.18	6.88	6.13	3.37	−4.16 to −2.58	Decreasing
Annual prevalence of the double burden of malnutrition (undernutrition + overweight) (%)																		
SAR	22.90	23.02	22.93	23.28	22.44	22.08	22.36	21.97	22.75	22.22	21.85	21.14	23.30	24.22	22.70	−0.04	−1.12 to −1.05	Stability
NSAR	21.85	22.04	21.87	22.04	21.37	21.65	21.64	21.25	21.66	21.25	21.14	20.66	22.98	23.73	21.47	0.07	−0.41 to −0.54	Stability
Brazil	22.20	22.35	22.20	22.41	21.69	21.78	21.85	21.46	21.96	21.52	21.34	20.79	23.06	23.86	21.75	0.01	−0.46 to −0.48	Stability

SAR: Brazilian semi-arid region; NSAR: non-semi-arid region; APC: annual percent change; 95%CI: 95% confidence interval.

Source: SISVAN Data (2008–2022).

Table 2 presents the prevalence for each stratum of nutritional status among children in the SAR, stratified by sex, ethnicity/skin color, and age group. A declining trend was observed for the prevalence of wasting, stunting, and consequently undernutrition; however, this pattern was not observed among Indigenous children, for whom the prevalence remained stable.

**Table 2:** Prevalence of overweight, wasting, stunting, undernutrition, and the double burden of malnutrition among children aged 5 to 9 years residing in the Brazilian semi-arid region, registered in the SISVAN, by sex, race/skin color, and age, Brazil, 2008–2022.

Parameter	Period															APC (%)	95%CI		Trend	
	2008	2009	2010	2011	2012	2013	2014	2015	2016	2017	2018	2019	2020	2021	2022					
	Annual prevalence of wasting (%)																			
Sex																				
Female	7.70	7.95	8.05	7.11	7.23	7.11	6.57	6.04	6.35	5.98	5.31	6.16	6.09	6.17	6.12	−2.12	−3.25 to −0.97		Decreasing	
Male	8.39	8.54	8.62	8.01	7.82	7.71	7.24	6.87	7.22	6.90	6.26	6.93	6.92	6.64	6.58	2.01	−2.61 to −1.41		Decreasing	
Race/skin color																				
Yellow	8.33	8.72	7.69	9.44	7.82	7.43	7.12	6.61	7.01	6.51	5.81	5.76	5.94	5.80	5.61	3.45	−4.05 to −2.84		Decreasing	
White	7.88	7.62	7.39	6.71	6.76	6.58	6.06	5.82	6.26	5.95	5.31	5.88	5.92	5.81	5.85	2.26	−3.25 to −1.26		Decreasing	
Indigenous	8.60	9.36	5.94	6.66	5.95	7.57	5.57	5.42	5.50	6.15	5.22	6.18	6.73	6.76	6.33	2.01	−4.09 to −0.11		Stability	
Mixed-race	8.96	8.56	8.10	7.57	7.48	7.52	6.73	6.21	6.63	6.38	5.87	6.77	6.64	6.58	6.54	2.29	−3.58 to −0.97		Decreasing	
Black	9.74	8.90	8.83	8.37	8.30	7.93	7.37	7.24	7.77	7.75	6.85	7.34	7.17	7.26	7.43	−1.95	−2.78 to −1.10		Decreasing	
Age (years)																				
05 to 06	8.31	8.35	8.41	7.80	7.75	7.53	7.10	6.76	7.07	6.75	6.02	6.97	7.03	6.90	6.94	−1.57	−2.51 to −0.63		Decreasing	
07 to 09	7.40	7.15	7.40	7.33	6.68	7.07	6.45	5.88	6.09	5.79	5.29	5.95	5.70	5.73	5.71	−2.23	−2.92 to −1.54		Decreasing	
05 to 09	8.05	8.25	8.34	7.76	7.52	7.41	6.84	6.36	6.67	6.33	5.68	6.48	6.43	6.37	6.33	−2.12	−3.08 to −1.15		Decreasing	
	Annual prevalence of overweight (%)																			
Sex																				
Female	21.59	22.04	22.91	24.34	24.57	25.07	27.04	27.94	28.52	28.33	28.64	27.37	30.58	32.12	30.19	2.61	2.06 to 3.16		Increasing	
Male	25.14	25.63	26.38	27.88	27.82	28.13	29.47	29.62	30.30	30.06	30.65	29.00	32.40	35.11	32.75	1.94	1.52 to 2.35		Increasing	
Race/skin color																				
Yellow	22.43	21.48	22.92	25.93	25.03	26.72	28.10	28.25	28.64	28.45	28.93	28.57	33.15	34.78	32.17	2.96	2.34 to 3.59		Increasing	
White	26.66	26.98	27.38	28.95	29.43	29.57	31.14	31.81	31.96	31.73	31.95	30.55	33.85	35.86	33.39	1.78	1.35 to 2.22		Increasing	
Indigenous	21.49	21.87	22.18	21.99	22.93	24.84	24.74	26.24	25.76	26.92	26.42	25.97	25.35	31.21	28.55	2.20	1.71 to 2.70		Increasing	
Mixed-race	23.65	23.81	24.22	25.47	25.52	25.81	27.13	28.18	28.90	28.44	28.70	27.27	30.47	32.72	30.77	2.08	1.66 to 2.50		Increasing	
Black	21.38	21.80	21.17	23.37	22.60	23.50	24.60	24.81	25.44	25.45	26.03	24.46	28.64	30.38	28.66	2.30	1.86 to 2.74		Increasing	
Age (years)																				
05 to 06	23.67	24.21	24.97	26.44	26.40	26.75	28.21	28.54	28.85	28.79	29.18	27.20	30.24	32.39	29.89	1.77	1.31 to 2.23		Increasing	
07 to 09	22.72	20.24	20.54	22.19	25.41	26.15	27.75	28.67	29.66	29.25	29.73	28.97	32.72	34.65	32.84	3.43	2.47 to 4.41		Increasing	
05 to 09	23.41	23.88	24.67	26.15	26.19	26.58	28.03	28.60	29.18	28.99	29.43	28.05	31.34	33.41	31.36	2.20	−1.79 to −2.62		Increasing	
	Annual prevalence of stunting (%)																			
Sex																				
Female	13.24	12.90	11.91	11.74	10.40	9.38	9.48	8.74	9.37	8.86	8.35	7.58	8.51	8.29	7.54	3.82	−4.79 to −2.85		Decreasing	
Male	15.39	14.84	13.75	13.50	11.93	11.00	10.39	9.36	10.14	9.52	8.95	7.97	9.26	9.11	7.92	−4.52	−5.62 to −3.39		Decreasing	
Race/skin color																				
Yellow	13.13	14.60	14.71	13.15	11.45	10.51	10.21	9.20	9.92	9.25	8.67	6.44	8.85	7.78	6.38	−5.35	−6.30 to −4.40		Decreasing	
White	12.34	12.72	11.53	11.29	10.03	9.26	9.10	8.43	9.14	8.80	8.34	7.54	8.41	8.35	7.32	−3.55	−4.53 to −2.55		Decreasing	
Indigenous	14.78	17.53	15.48	14.98	12.24	12.08	12.02	12.06	12.58	15.80	12.33	10.91	15.02	15.03	14.05	−0.68	−2.70 to 1.39		Stability	
Mixed-race	14.16	14.23	13.12	12.83	11.30	10.18	9.85	8.97	9.58	9.03	8.58	8.06	8.93	8.81	8.01	−4.05	−5.35 to −2.74		Decreasing	
Black	14.30	14.71	13.00	13.21	11.74	11.18	9.97	9.22	9.78	9.53	8.83	7.29	9.33	8.30	7.64	−4.55	−5.42 to −3.68		Decreasing	
Age (years)																				
05 to 06	13.81	13.94	12.82	12.58	10.97	9.81	9.34	8.69	9.60	8.96	8.58	7.93	8.75	8.77	8.08	−3.84	−5.29 to −2.37		Decreasing	
07 to 09	15.71	13.44	13.14	13.49	11.87	11.15	10.64	9.34	9.74	9.31	8.59	7.55	8.90	8.49	7.35	−4.81	−5.58 to −4.03		Decreasing	
05 to 09	14.34	13.90	12.84	12.64	11.16	10.18	9.85	8.98	9.66	9.11	8.58	7.75	8.82	8.65	7.71	4.25	−5.33 to −3.15		Decreasing	
	Annual prevalence of undernutrition (%)																			
Sex																				
Female	10.47	10.43	9.98	9.62	8.81	8.24	8.03	7.39	7.86	7.42	6.83	6.87	7.30	7.23	6.83	−3.11	−4.11 to −2.10		Decreasing	
Male	11.89	11.69	11.18	10.75	9.87	9.36	8.81	8.11	8.68	8.21	7.60	7.45	8.09	7.87	7.25	−3.49	−4.43 to −2.55		Decreasing	
Race/skin color																				
Yellow	10.73	11.66	11.37	11.19	9.63	8.97	8.67	7.90	8.47	7.88	7.24	6.10	7.39	6.79	6.00	−4.56	−5.31 to −3.79		Decreasing	
White	10.11	10.17	9.60	9.14	8.40	7.92	7.58	7.13	7.70	7.37	6.83	6.71	7.17	7.08	6.59	−3.02	−4.03 to −2.01		Decreasing	
Indigenous	11.68	13.44	11.09	11.18	9.10	9.83	8.79	8.74	9.04	10.98	8.78	8.54	10.87	10.90	10.19	−1.12	−3.05 to 0.85		Stability	
Mixed-race	11.56	11.39	10.75	10.34	9.39	8.85	8.29	7.59	8.10	7.70	7.23	7.42	7.78	7.69	7.28	−3.30	−4.63 to −1.95		Decreasing	
Black	12.02	11.81	10.99	11.00	10.02	9.56	8.67	8.23	8.77	8.64	7.84	7.31	8.25	7.78	7.54	−3.39	−4.29 to −2.48		Decreasing	
Age (years)																				
05 to 06	11.06	11.15	10.62	10.19	9.36	8.67	8.22	7.73	8.34	7.85	7.30	7.45	7.89	7.84	7.11	−2.83	−4.10 to −1.55		Decreasing	
07 to 09	11.55	10.29	10.26	10.40	9.27	9.11	8.54	7.61	7.92	7.55	6.94	6.75	7.30	7.11	6.53	−3.86	−4.58 to −3.14		Decreasing	
05 to 09	11.19	11.08	10.59	10.20	9.34	8.79	8.35	7.67	8.16	7.72	7.13	7.11	7.63	7.11	7.02	3.36	−4.40 to −2.30		Decreasing	
	Annual prevalence of the double burden of malnutrition (undernutrition + overweight) (%)																			
Sex																				
Female	21.26	21.45	21.44	21.8	21.1	20.78	21.55	21.36	22.12	21.59	21.15	20.56	22.58	23.29	21.92	0.30	−0.09 to 0.69		Stability	
Male	24.46	24.51	24.38	24.7	23.78	23.43	23.55	22.93	23.83	23.24	22.93	21.95	24.29	25.43	23.63	−0.23	−0.76 to 0.30		Stability	
Race/Skin color																				
Yellow	21.95	22.43	22.84	24.16	22.15	22.33	22.72	22.02	22.79	22.11	21.7	20.39	23.97	24.18	22.08	−0.01	−0.56 to 0.54		Stability	
White	23.45	23.67	23.3	23.63	23.13	22.71	23.15	23.03	23.68	23.24	22.8	21.98	24.09	25.01	23.28	0.06	−0.33 to 0.45		Stability	
Indigenous	22.44	24.38	22.18	22.18	20.56	22.25	21.16	21.86	21.92	24.43	21.98	21.53	23.55	26.5	24.47	0.61	−0.28 to 1.52		Stability	
Mixed-race	23.39	23.3	22.86	23.08	22.15	21.76	21.86	21.68	22.56	21.92	21.58	21.05	23.02	24.05	22.66	−0.16	−0.78 to 0.46		Stability	
Black	22.71	22.71	21.58	22.69	21.33	21.3	20.97	20.64	21.49	21.37	20.85	19.55	22.57	22.97	21.87	−0.22	−0.85 to 0.41		Stability	
Age (years)																				
05 to 06	22.89	23.26	23.1	23.41	22.56	22.05	22.32	22	22.76	22.25	21.89	21.05	23.01	24.03	22.45	−0.15	−0.62 to 0.32		Stability	
07 to 09	22.91	20.42	20.57	21.53	21.99	22.19	22.42	21.94	22.75	22.18	21.81	21.23	23.66	24.44	22.95	0.61	0.12 to 1.10		Increasing	
05 to 09	22.90	23.02	22.93	23.28	22.44	22.08	22.36	21.97	22.75	22.22	21.85	21.14	23.3	24.22	22.70	−0.04	−0.54 to 0.45		Stability	

APC: annual percent change; 95%CI: 95% confidence interval.

Source: SISVAN Data (2008–2022).

The prevalence of overweight showed an increasing trend across all categories analyzed. Regarding DBM, the 7–9-year-old age group was the only category showing a rising trend. Although the other categories exhibited stable trends, high DBM prevalence persisted throughout the study period in the SAR (Table 2).

**Figure 2:**
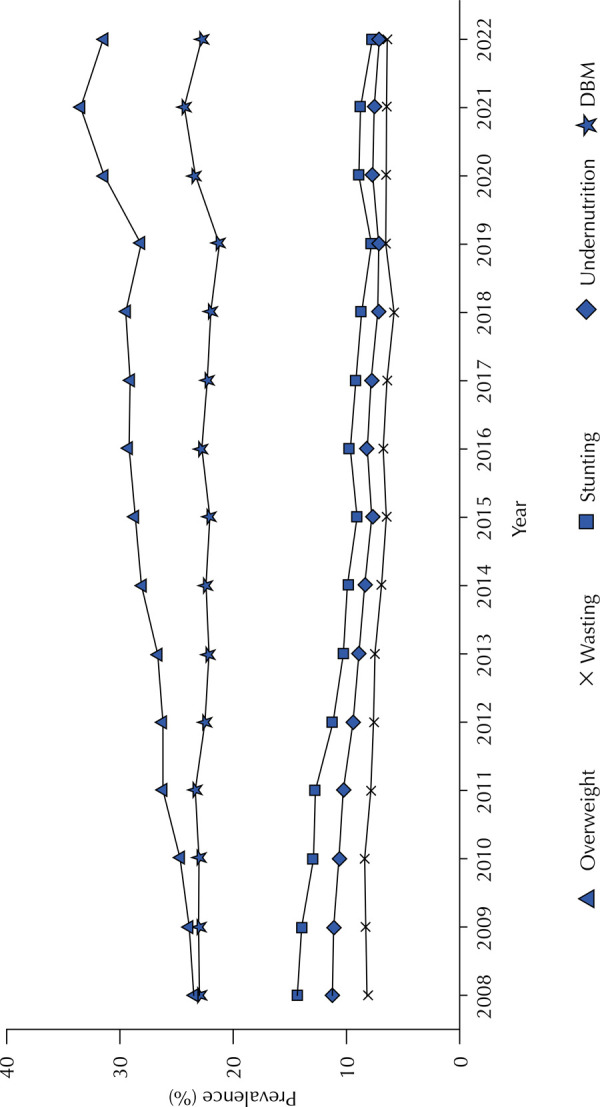
Temporal evolution of the prevalence of overweight, wasting, stunting, undernutrition, and the double burden of malnutrition among children aged 5 to 9 years residing in the Brazilian semi-arid region, registered in the SISVAN, Brazil, 2008–2022.

In Figure 2, the high prevalence of DBM in the SAR becomes even more evident. Over the time series analyzed, DBM prevalence approached that of overweight, while the prevalence of wasting, stunting, and undernutrition declined.

DBM: double burden of malnutrition.

Source: SISVAN Data (2008–2022).

**Figure 3:**
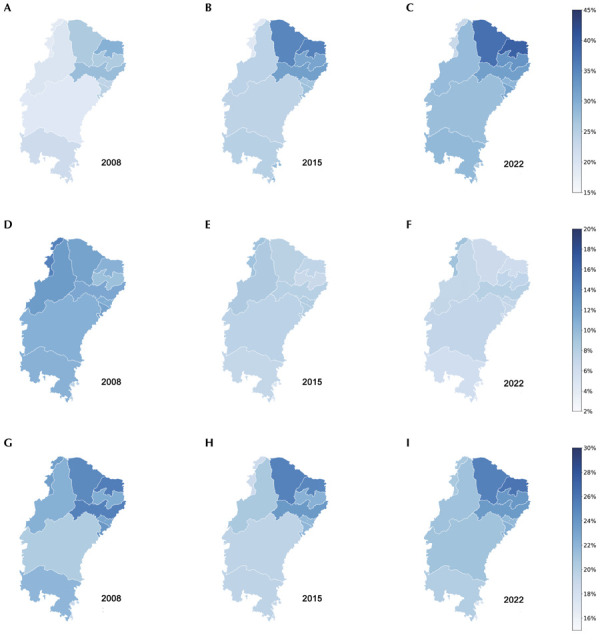
Temporal evolution of the prevalence (%) of overweight (A,B,C), undernutrition (D,E,F), and the double burden of malnutrition (G,H,I) among children aged 5 to 9 years residing in the Brazilian semi-arid region, according to the federative units/states, Brazil, 2008, 2015, and 2022.

Source: SISVAN Data (2008, 2015, 2022).

Regarding spatial distribution, there was an increase in overweight prevalence in the SAR from 2008 to 2022—from 23.41% to 31.36%—with higher prevalence observed in the northern states of the region, such as Ceará and Rio Grande do Norte, where darker colors on the map indicate higher rates. In contrast, undernutrition prevalence decreased throughout the SAR, from 11.19% in 2008 to 7.02% in 2022. As for DBM, prevalence declined from 2008 (22.90%) to 2015 (21.97%), an increase from 2015 to 2022 (22.70%), returning to levels similar to those observed in 2008 (Figure 3).

## DISCUSSION

The results of this study show a high prevalence of the double burden of malnutrition (DBM) in the semi-arid region (SAR) (22.70%) and also in the non-semi-arid region (NSAR) (21.47%) in 2022. The temporal evolution of DBM prevalence in the SAR showed an increasing trend among children aged 7 to 9 years and stability among those under 7 years of age. This followed the pattern of overweight prevalence, which increased across all regions, sexes, age groups, and race/skin color categories. Conversely, undernutrition showed a decreasing or stable trend across regions, except among the Indigenous population in the SAR. DBM is a complex phenomenon, closely linked to low socioeconomic levels and the rapid nutritional transition taking place in the SAR. This transition can be partly explained by the reduced consumption of natural foods, which have been replaced by more processed foods rich in fats and simple sugars in the child population^22^.

The increasing trend in DBM among children aged 7 to 9 years, the group with the highest APC in overweight prevalence, is a matter of concern. In this scenario, both individuals and society experience the consequences of undernutrition and overweight simultaneously^1^. De Sanctis et al.^23^ demonstrated that child undernutrition, caused by low caloric intake and genetic factors, alters energy balance, oxidation, and fat accumulation later in life, thereby increasing the predisposition to obesity. Wells et al.^1^ corroborate these findings, linking child undernutrition to changes in the gut microbiota and organs such as the liver and kidneys, as well as to an increased risk of overweight^1,23^. Similar to undernutrition, overweight affects insulin metabolism and satiety control via leptin action in the hypothalamus, shaping food preferences and the gut microbiota^1,23^. This condition increases the risk of developing noncommunicable chronic diseases (NCDs) and impacts the health of future generations, as both conditions have intergenerational effects^1,23^. These findings underscore the need to view undernutrition and overweight as coexisting conditions that arise from shared determinants and have interconnected consequences^1^.

The APCs of nutritional status indicators among children in the SAR were more pronounced over the study period for both overweight (APC = 2.20%; 95%CI: 1.79 to 2.62) and undernutrition (APC = −3.36%; 95%CI: −4.40 to −2.30). Santos and Fontão^2^ highlighted the relationship between Brazil’s nutritional transition and changes in dietary patterns, observing an average 5.5% increase in ultra-processed food consumption over 10 years (2008–2018). They emphasize that this change is most evident in socioeconomically vulnerable regions, where access to healthy foods is limited. Popkin et al.^3^ also reported that DBM has increased by more than one percentage point per year in low- and middle-income countries, driven by the rise in overweight. The social inequality affecting the SAR population is evident when analyzing per capita GDP distribution. A 2017 study showed that just over 200 municipalities in the SAR had per capita income above the minimum wage at that time^18^. These factors are directly linked to food insecurity and nutritional deficiencies, as they significantly limit access to healthy foods^18^. These results highlight the need for stronger public health interventions in the SAR, which continues to lack adequate support, considering that overweight and undernutrition share common social and health determinants^22^.

Another point of concern raised by this study is the racial disparity observed for each condition. Indigenous children showed a lower APC for the prevalence of wasting, stunting, and undernutrition, with a stable trend, while the prevalence of overweight increased, indicating both a nutritional transition and insufficient public support in this group, perpetuating malnutrition in all its forms. The Indigenous population is among the most affected by social inequality in Brazil, influenced by factors such as land conflicts, limited access to healthcare services—due to the distance from the basic health units and delayed care-seeking—low income, and cultural barriers, all of which hinder nutritional monitoring^24^.

The accelerated nutritional transition, characterized by the overlap of public health issues and the coexistence of nutritional imbalances within the same population, has been a concern since Brazil’s so-called “nutritional call” about 20 years ago^25^. Currently, this concern is confirmed by the maps and figures in this study, which reveal the influence of decreasing undernutrition (from 11.19% in 2008 to 7.02% in 2022 in the SAR) and increasing overweight (from 23.41% in 2008 to 31.36% in 2022 in the SAR) on the temporal evolution of DBM. These findings demonstrate the rapid nutritional transition in the SAR, where the rate of increase in overweight was twice as fast as the rate of decline in undernutrition. Another indicator of this moderately faster transition in the SAR compared to the NSAR is the prevalence of stunting, with an APC of −4.25% (95%CI: -5.33 to −3.15) in the SAR and −3.85% (95%CI: −4.63 to −3.06) in the NSAR. Similarly, overweight prevalence also increased faster in the SAR, with an APC of 2.20% (95%CI: 1.79 to 2.62), indicating that the region—historically burdened by undernutrition —now faces a rapid rise in overweight and obesity. Similarly, Rocha^26^ (2016), using cross-sectional population-based surveys from the SAR from 1987 to 2007, also found that acute undernutrition decreased drastically, from 12.4% (95%CI: 11.4 to 13.4) to 4.3% (95%CI: 3.3 to 5.4), while child obesity increased alarmingly, by almost 240%, with a temporal trend of 12.01%. Silva^27^ (2019) conducted a longitudinal analysis of Brazilian municipalities participating in the *Bolsa Família* Program, showing that the advanced stage of Brazil’s nutritional transition was characterized by an increase in overweight (from 17.2% to 18.4%), both alone and coexisting with undernutrition (i.e., DBM), particularly among socioeconomically vulnerable children. The study also associated DBM prevalence with socioeconomic factors such as per capita GDP, expected years of schooling, unemployment rate, and household crowding^27^.

Despite the complex context of socioeconomic inequality in Brazil, inclusive social policies implemented since 2003, such as the Bolsa Família Program, have contributed significantly to reducing food insecurity and improving dietary patterns^14,24^. Improvements in socioeconomic conditions may have led to better child nutrition and height; however, they may also have contributed to greater availability of calorie-dense and ultra-processed foods, thus driving the increase in overweight observed in this study. Although socioeconomic advances occurred, the recent political and economic crisis haves strongly impacted the country’s food and nutrition security, particularly among the most vulnerable populations. This is reflected in increased unemployment, inflation, and food insecurity—all of which influence DBM^26,28,29^. In this context, for health professionals and policymakers to act effectively, socioeconomic, cultural, political, and health factors must be understood in an integrated manner.^28^ These findings highlight the importance of public policies toward reducing social inequality and improving the nutritional status of the most vulnerable children.

This study shows limitations inherent to the use of secondary data, such as the limited quality and low coverage of SISVAN in some regions^11,30^. A recent study evaluating SISVAN coverage from 2008 to 2021 found that since its implementation in 2008, SISVAN-Web coverage increased across all regions and age groups until 2019^11,30^. However, in 2020, the first year of the covid-19 pandemic, coverage declined substantially, recovering in 2021 for the 0–4 and 5–9-year-old groups^11,30^.

Despite these limitations, this study reinforces the importance of nutritional monitoring and early surveillance, as well as the need for government programs that effectively target vulnerable populations. Such programs help prevent and manage malnutrition while contributing to hunger eradication and improved quality of life, especially among the most vulnerable. Moreover, this study stands out for its novelty, given the scarcity of research on the extent of nutritional imbalances (undernutrition and overweight) coexisting in this territory. While progress has been made in studying undernutrition based on weight and height indices, we were unable to assess all its forms, such as micronutrient deficiencies. Therefore, further studies are needed to investigate undernutrition in its full scope.

In conclusion, the double burden of malnutrition, with the simultaneous presence of undernutrition and overweight, remains an even greater challenge in a continental country like Brazil—particularly in the semi-arid region, marked by low socioeconomic levels and an accelerated nutritional transition. Despite advances in public health, it is essential to prioritize the Food and Nutrition Agenda by focusing on the early stages of life. Health promotion and malnutrition prevention strategies should address all forms of malnutrition, especially among children and Indigenous populations.

## Data Availability

Raw data of the article are available upon request to the corresponding author.

## Footnotes

a Available from: https://sisaps.saude.gov.br/sisvan/relatoriopublico/
