# Supplementary material for: Spatio-temporal analysis of the double burden of malnutrition in children from the Brazilian semi-arid region
Source: Rev Saude Publica. 2025 Dec 8;59:e40. doi: 10.11606/s1518-8787.2025059006605 (PMC12674690; doi:10.11606/s1518-8787.2025059006605)
Supplement: Translation

# Translation: Análise espaço-temporal da dupla carga de má nutrição em crianças do semiárido brasileiro

**Authors:** Maisa Póvoa de Oliveira, Cíntia Pereira Donateli, Daniela Mayumi Usuda Prado Rocha, Helen Hermana Miranda Hermsdorff

**Affiliations:** I Universidade Federal de Viçosa. Departamento de Nutrição e Saúde. Viçosa, MG, Brasil; II Universidade Federal de Viçosa. Instituto de Políticas Públicas e Desenvolvimento Sustentável. Viçosa, MG, Brasil

**Keywords:** Estado Nutricional, Nutrição da Criança, Atenção Primária à Saúde, Análise de Dados Secundários, Prevalência, Mapeamento Geográfico

## Abstract

**OBJETIVO:**

Analisar a tendência temporal da dupla carga de má nutrição no semiárido brasileiro e no não semiárido brasileiro, e avaliar sua distribuição espacial no semiárido brasileiro em crianças de 5 a 9 anos, no período de 2008 a 2022.

**MÉTODOS:**

Estudo ecológico de série temporal a partir dos relatórios públicos, utilizando macrodados de índice de massa corporal para idade e altura para idade do Sistema de Vigilância Alimentar e Nutricional (Sisvan). Para análise temporal das prevalências de emaciação, de excesso de peso, de déficit de altura, de desnutrição e da dupla carga de má nutrição nas regiões do semiárido brasileiro e não semiárido brasileiro, utilizaram-se modelos de regressão de Prais-Winsten, com cálculo da variação percentual anual (APC%) de 2008 a 2022. Para avaliação da distribuição espacial na região do semiárido brasileiro, foram construídos mapas de evolução temporal das prevalências de excesso de peso, desnutrição e dupla carga de má nutrição.

**RESULTADOS:**

De 2008 a 2022, 48,7 milhões de crianças de 5 a 9 anos foram registradas no Sisvan-Web para os índices de massa corporal para idade e altura para idade. Aproximadamente, 28,1% dessas residiam no semiárido brasileiro. A prevalência de dupla carga de má nutrição no semiárido brasileiro foi superior a 20%, com tendência crescente para crianças de 7 a 9 anos (APC = 0,61%; p < 0,05). O excesso de peso seguiu tendência crescente, enquanto a desnutrição diminuiu em todas as estratificações, exceto entre a população indígena, que apresentou estabilidade (APC = -1,12%; p > 0,05). As APC no semiárido brasileiro foram maiores do que na região do não semiárido.

**CONCLUSÃO:**

O estudo apresenta uma alta prevalência da dupla carga de má nutrição no semiárido brasileiro, especialmente entre as crianças de 7 a 9 anos. Dessa forma, é crucial promover ações de saúde e prevenção da má nutrição, fortalecendo políticas já existentes e priorizando as populações mais vulneráveis.

## INTRODUÇÃO

Em populações que enfrentam vulnerabilidade socioeconômica, o aumento da prevalência de excesso de peso tem se apresentado de forma acelerada, enquanto a queda de desnutrição tem ocorrido de forma lenta^1^. Esse fenômeno resulta na presença da dupla carga de má nutrição (DCMN), definida pela coexistência de excesso de peso e desnutrição em populações, famílias ou indivíduos^1^.

A presença desse duplo ônus da má nutrição caracteriza-se como um importante problema de saúde pública^4^. Globalmente, estima-se que 190 milhões de crianças e adolescentes entre 5 e 19 anos apresentavam baixo peso, enquanto 160 milhões viviam com excesso de peso no ano de 2022, evidenciando assim uma prevalência de 17,8% da DCMN a nível mundial^4^. No Brasil, o cenário também é preocupante, com estimativas de que 6,4 milhões de crianças tenham excesso de peso e 3,1 milhões já evoluíram para obesidade^5^. Ao mesmo tempo, a prevalência de desnutrição, apesar de ter sofrido queda importante nas últimas décadas, apresentou um salto a partir do ano de 2018, chegando a 5,3% no ano de 2021 entre crianças e adolescentes de 0 a 19 anos^6^.

O excesso de peso infantil, além de ser um fator de risco para o desenvolvimento precoce de outras doenças crônicas não transmissíveis (DCNT) – como diabetes tipo 2, doenças cardiovasculares e câncer –, está relacionado à redução da mobilidade, dores articulares e transtornos psicológicos influenciados pelo estigma social e *bullying*, como depressão e transtornos alimentares^7,8^. Por sua vez, a desnutrição crônica pode gerar danos a curto e médio prazo, por exemplo, atrasos no crescimento e no desenvolvimento cognitivo das crianças, afetando as habilidades motoras e de aprendizado; enfraquecimento do sistema imunológico; e danos futuros como a predisposição a DCNT – assim como o excesso de peso – e, em casos mais graves, pode levar ao óbito^9^. Diante disso, a presença da DCMN na infância pode trazer consequências para toda a população, no âmbito da saúde e também no desenvolvimento socioeconômico, já que afeta o desempenho escolar e ocasiona a redução da produtividade e capital humano, o aumento da morbimortalidade e a perda na qualidade de vida^10,11^.

Nesse cenário de DCMN, a avaliação e o monitoramento do estado nutricional infantil, principalmente em regiões de maior vulnerabilidade, como o semiárido brasileiro (SB), tornam-se uma importante ferramenta para a identificação de padrões epidemiológicos, determinantes sociais e agravos nutricionais tanto precoces quanto futuros. O SB, semiárido mais populoso do mundo com aproximadamente 31 milhões de pessoas^12^, é uma região marcada por alta incidência solar e grande variabilidade de precipitação pluviométrica, tendo, portanto, grandes períodos de seca, que influenciam na produção e distribuição de alimentos^13,14^. Além da vulnerabilidade climática, a região enfrenta vulnerabilidades socioeconômicas, como escassez de renda, educação e saneamento^15^.

Ademais, carece na literatura informações sobre o estado nutricional de crianças do SB, pois é limitado de dados ao longo do tempo. Essa ausência de evidências prejudica a compreensão como um todo das particularidades e desafios enfrentados pelas crianças dessa região que possui características socioeconômicas, ambientais e climáticas específicas. Conhecer e monitorar o estado nutricional da população infantil do SB, bem como esse se posiciona em relação a outras regiões é crucial para definição de prioridades. No Brasil, esse monitoramento é realizado pela Atenção Primária à Saúde (APS) a partir das medidas antropométricas registradas no Sistema de Vigilância Alimentar e Nutricional (Sisvan)^16^. Para crianças de 5 a 9 anos, é possível acessar relatórios com informações sobre os índices: peso para idade, altura para idade (A/I) e índice de massa corporal para idade (IMC/I)^16^.

Diante da necessidade de pesquisas que apresentem a situação da DCMN em crianças do SB, este estudo teve como objetivo analisar a tendência temporal da DCMN no SB e no não semiárido brasileiro (NSB) e avaliar sua distribuição espacial no SB em crianças de 5 a 9 anos, no período de 2008 a 2022.

## MÉTODOS

### Delineamento do Estudo e Fonte de Dados

Trata-se de um estudo ecológico de série temporal, com base em dados secundários disponíveis no Sisvan e agregados por municípios, que buscou avaliar a prevalência de DCMN, definida pela coexistência de desnutrição e excesso de peso, em crianças de 5 a 9 anos, residentes na região do SB, entre 2008 e 2022.

O Sisvan fornece dados relativos às ações de vigilância alimentar e nutricional quanto a indivíduos atendidos na APS, cujo registro foi realizado pelos profissionais das unidades básicas de saúde, durante o período de ações no e-SUS Atenção Básica e no Sistema de Gestão do Programa Bolsa Família, que migram periodicamente, de forma automática, para a plataforma do Sisvan^16,17^.

Assim, dados antropométricos do estado nutricional das crianças em relação ao período de 2008 a 2022 e agregados por município foram extraídos por meio dos relatórios consolidados de acesso público e irrestrito, disponíveis para download no endereço eletrônico do Sisvan-Web^a^.

### Caracterização Geográfica

O estudo teve como unidades de análise os dados agregados por município do Brasil e as regiões do SB e NSB. O SB foi definido de acordo com a Resolução do Conselho Deliberativo da Superintendência do Desenvolvimento do Nordeste nº 150, de 13 de dezembro de 2021, que discrimina: dos 5.570 municípios brasileiros, 1.477 são do SB, abrangendo assim 11 estados brasileiros: Maranhão, Piauí, Ceará, Rio Grande do Norte, Paraíba, Pernambuco, Alagoas, Sergipe, Bahia, Minas Gerais e Espírito Santo^18^(Figura 1). Já na região definida como NSB, enquadraram-se os 4.093 municípios restantes.

**Figura 1:** Demarcação da região do semiárido no território brasileiro (A) e demarcação das Unidades Federativas que compõem o semiárido brasileiro (B).

MA: Maranhão; CE: Ceará; RN: Rio Grande do Norte; PB: Paraíba; PI: Piauí; PE: Pernambuco; AL: Alagoas; SE: Sergipe; BA: Bahia; MG: Minas Gerais; ES: Espírito Santo.

Fonte: CONDEL/SUDENE,2021^18^.

### Participantes

Neste estudo, foram incluídas todas as crianças de 5 a 9 anos de idade, de ambos os sexos, com registro das medidas antropométricas no Sisvan, para cada município-ano, no período de 2008 a 2022. Os dados, então, foram agrupados para abranger as análises do SB e NSB.

### Análise de Dados

A avaliação do estado nutricional seguiu o padrão de crescimento da Organização Mundial da Saúde (2007). A partir do índice IMC/I^12^, as crianças foram classificadas em: sobrepeso (> escore-z +1 e ≤ escore-z +2), obesidade (> escore-z +2 e ≤ escore-z +3) e obesidade grave (> escore-z +3). Já a classificação de magreza com valores críticos entre (≥ escore-z -3 e < escore-z -2) e magreza acentuada (< escore-z -3) pelo índice IMC/I^16^. Quanto ao índice A/I, as crianças foram classificadas em altura muito baixa para a idade (escore-z < -3) e baixa altura para a idade (escore-z ≥ -3 e escore-z < -2)^16^. Partindo das classificações desses índices, calcularam-se as prevalências dos desfechos antropométricos de interesse, a seguir.

A prevalência de emaciação foi classificada a partir do agrupamento entre magreza e magreza acentuada, utilizando a fórmula:


$$\text{ prevalência de emaciação }(\%)=\frac{\mathrm{magreza}(n)+\text{ magreza acentuada }(n)}{\text{ total avaliado por }\mathrm{IMC}/I(n)}\times 100$$

Para o estudo, a prevalência de excesso de peso foi calculada com o agrupamento das classificações de sobrepeso, obesidade e obesidade grave como excesso de peso^19^, conforme a seguinte fórmula:


$$\text{ prevalência de excesso de peso }(\%)=\frac{\mathrm{sobrepeso}(n)+\text{ obesidade }(n)+\text{ obesidade grave }(n)}{\text{ total avaliado por }\mathrm{IMC}/I(n)}\times 100$$

A prevalência de déficit de altura foi representada pelas classificações de altura muito baixa para a idade e baixa altura para a idade, com a fórmula:


$$\text{ prevalência de déficit de altura }(\%)=\frac{\begin{array}{c} \text{ altura muito baixa para a idade }(n)+\\ \text{ baixa altura para a idade }(n) \end{array}}{\text{ total avaliado por }A/I(n)}\times 100$$

Em seguida, a prevalência de desnutrição foi calculada a partir dos indicadores de desnutrição aguda (emaciação) ou pregressa (déficit de altura)^16,19^, a partir da fórmula:


$$\text{ prevalência de déficit de altura }(\%)=\frac{\begin{array}{c} \text{ altura muito baixa para a idade }(n)+\\ \text{ baixa altura para a idade }(n) \end{array}}{\text{ total avaliado por }A/I(n)}\times 100$$

A prevalência da DCMN, foco deste estudo, foi definida como a presença simultânea de desnutrição (emaciação e déficit de altura) e de excesso de peso (sobrepeso, obesidade e obesidade grave)^1,10,19^, de acordo com a fórmula:


$$\text{ prevalência de desnutrição }(\%)=\frac{\text{ emaciação }(n)+\text{ déficit de altura }(n)}{\text{ total avaliado por }\mathrm{IMC}/I(n)+}\times 100$$

A tendência temporal das prevalências do estado nutricional, conforme a delimitação territorial do SB, NSB e o Brasil como um todo para cada ano de acompanhamento, foram analisadas utilizando-se de modelos de regressão de Prais-Winsten, abordagem recomendada a estudos ecológicos para controlar a autocorreção dos resíduos de regressão entre os anos analisados20. Ainda, analisaram-se esses dados de acordo com a estratificação por sexo, raça/cor e faixa etária, para o SB. A variação percentual anual (APC) e seus respectivos intervalos de confiança (IC95%) em porcentagem foram calculados a partir da seguinte fórmula, (-1 + 10^β) x 100, em que β são os valores do coeficiente resultante da regressão de Prais-Winsten. P-valores não significantes (p ≥ 0,05) indicaram tendência de estabilidade, e p-valores significantes (p < 0,05), tendência crescente ou decrescente, conforme a variação positiva ou negativa, respectivamente^20^. As análises dos dados realizaram-se no software R, versão 4.3.3.

Mapas para a representação da delimitação do SB18 foram construídos por meio das bibliotecas GeoPandas e Matplotlib do Python versão 3.11.5, sendo utilizada a base cartográfica, no formato shapefile, referente aos limites dos municípios, das Unidades Federativas do Brasil e do Instituto Brasileiro de Geografia e Estatística^21^. Também montaram-se mapas para representar a evolução na distribuição espacial das prevalências de excesso de peso, desnutrição e DCMN no SB, nos anos de 2008, 2015 e 2022.

### Aspectos Éticos

O estudo utilizou dados secundários de domínio público, estando dispensado de avaliação por parte de um comitê de ética em pesquisas em seres humanos.

## RESULTADOS

No Brasil, durante o período de 2008 a 2022, foram identificados 48.714.371 registros de crianças de 5 a 9 anos no Sisvan-Web para o índice de IMC/I e 48.712.533 registros de crianças da mesma faixa etária para o índice de A/I. Destas, 13.704.166 (28,13%) e 13.701.835 (28,13%), respectivamente, residiam na região do SB.

As prevalências de emaciação, déficit de altura e desnutrição apresentaram tendências decrescentes em ambas as regiões analisadas. Já a prevalência de excesso de peso mostrou tendência crescente em todas as regiões, porém com maior variação percentual (APC = 2,20%; IC95% 1,79 a 2,62) no SB. A prevalência de DCMN indicou tendência de estabilidade tanto no SB quanto no NSB (Tabela 1).

**Tabela 1:** Tendência temporal da prevalência da dupla carga de má nutrição em crianças de 5 a 9 anos, cadastrados no Sistema de Vigilância Alimentar e Nutricional por região, Brasil, 2008-2022.

Variáveis	Período															APC (%)	IC95%	Tendência
	2008	2009	2010	2011	2012	2013	2014	2015	2016	2017	2018	2019	2020	2021	2022			
Prevalência anual de emaciação (%)																		
SB	8,05	8,25	8,34	7,76	7,52	7,41	6,84	6,36	6,67	6,33	5,68	6,48	6,43	6,37	6,33	-2,12	-3,08 a -1,15	Decrescente
NSB	6,37	6,36	6,50	6,22	5,93	6,04	5,77	5,10	5,19	5,07	4,68	5,41	5,26	5,08	4,96	-2,02	-2,82 a -1,21	Decrescente
Brasil	6,92	6,95	7,06	6,69	6,40	6,45	6,09	5,46	5,60	5,43	4,97	5,70	5,56	5,41	5,28	-2,20	-3,01 a -1,38	Decrescente
Prevalência anual de excesso de peso (%)																		
SB	23,41	23,88	24,67	26,15	26,19	26,58	28,03	28,60	29,18	28,99	29,43	28,05	31,34	33,41	31,36	2,20	1,79 a 2,62	Crescente
NSB	24,51	25,16	25,45	26,56	26,95	27,37	27,60	28,79	29,22	28,80	29,27	28,18	31,91	34,15	31,23	1,91	1,51 a 2,32	Crescente
Brasil	24,15	24,76	25,21	26,44	26,72	27,13	27,73	28,74	29,21	28,86	29,31	28,15	31,76	33,96	31,26	2,01	1,61 a 2,40	Crescente
Prevalência anual de déficit de altura (%)																		
SB	14,34	13,90	12,84	12,64	11,16	10,18	9,85	8,98	9,66	9,11	8,58	7,75	8,82	8,65	7,71	-4,25	-5,33 a -3,15	Decrescente
NSB	12,81	12,55	11,79	11,29	9,86	9,89	9,90	8,61	8,90	8,63	8,33	7,72	8,79	8,23	6,77	-3,85	-4,63 a -3,06	Decrescente
Brasil	13,31	12,97	12,11	11,69	10,25	9,98	9,89	8,72	9,11	8,76	8,40	7,73	8,80	8,34	6,99	-4,03	-4,86 a -3,20	Decrescente
Prevalência anual de desnutrição (emaciação + déficit de altura) (%)																		
SB	11,19	11,08	10,59	10,20	9,34	8,79	8,35	7,67	8,16	7,72	7,13	7,11	7,63	7,51	7,02	-3,36	-4,40 a -2,30	Decrescente
NSB	9,59	9,45	9,14	8,75	7,89	7,97	7,84	6,85	7,05	6,85	6,50	6,57	7,03	6,65	5,87	-3,21	-3,95 a -2,47	Decrescente
Brasil	10,12	9,96	9,58	9,19	8,32	8,22	7,99	7,09	7,36	7,09	6,68	6,71	7,18	6,88	6,13	-3,37	-4,16 a -2,58	Decrescente
Prevalência anual de dupla carga de má nutrição (desnutrição + excesso de peso) (%)																		
SB	22,90	23,02	22,93	23,28	22,44	22,08	22,36	21,97	22,75	22,22	21,85	21,14	23,30	24,22	22,70	-0,04	-1,12 a 1,05	Estabilidade
NSB	21,85	22,04	21,87	22,04	21,37	21,65	21,64	21,25	21,66	21,25	21,14	20,66	22,98	23,73	21,47	0,07	-0,41 a 0,54	Estabilidade
Brasil	22,20	22,35	22,20	22,41	21,69	21,78	21,85	21,46	21,96	21,52	21,34	20,79	23,06	23,86	21,75	0,01	-0,46 a 0,48	Estabilidade

SB: semiárido brasileiro; NSB: não semiárido brasileiro; APC: variação percentual anual; IC95%: intervalo de confiança de 95%.

Fonte: dados do Sisvan (2008–2022).

A Tabela 2 retrata as prevalências para cada um dos estratos do estado nutricional das crianças do SB, especificamente, quanto ao sexo, raça/cor e faixa etária. Observa-se que para as prevalências de emaciação, déficit de altura e consequentemente da desnutrição, há uma tendência decrescente. No entanto, o mesmo não se observa para a categoria indígena. Para a população indígena houve uma tendência de estabilidade dessas prevalências.

**Tabela 2:** Prevalência do excesso de peso, emaciação, déficit de altura, desnutrição e dupla carga de má nutrição em crianças de 5 a 9 anos, residentes no semiárido brasileiro, cadastradas no Sistema de Vigilância Alimentar e Nutricional, por sexo, raça/cor e idade, Brasil, 2008–2022.

Variáveis	Período															APC (%)	IC95%		Tendência	
	2008	2009	2010	2011	2012	2013	2014	2015	2016	2017	2018	2019	2020	2021	2022					
	Prevalência anual de emaciação (%)																			
Sexo																				
Feminino	7,70	7,95	8,05	7,51	7,23	7,11	6,57	6,04	6,35	5,98	5,31	6,16	6,09	6,17	6,12	-2,12	-3,25 a -0,97		Decrescente	
Masculino	8,39	8,54	8,62	8,01	7,82	7,71	7,24	6,87	7,22	6,90	6,26	6,93	6,92	6,64	6,58	-2,01	-2,61 a -1,41		Decrescente	
Raça/cor																				
Amarela	8,33	8,72	7,69	9,44	7,82	7,43	7,12	6,61	7,01	6,51	5,81	5,76	5,94	5,80	5,61	-3,45	-4,05 a -2,84		Decrescente	
Branca	7,88	7,62	7,39	6,71	6,76	6,58	6,06	5,82	6,26	5,95	5,31	5,88	5,92	5,81	5,85	-2,26	-3,25 a -1,26		Decrescente	
Indígena	8,60	9,36	5,94	6,66	5,95	7,57	5,57	5,42	5,50	6,15	5,22	6,18	6,73	6,76	6,33	-2,01	-4,09 a 0,11		Estabilidade	
Parda	8,96	8,56	8,10	7,57	7,48	7,52	6,73	6,21	6,63	6,38	5,87	6,77	6,64	6,58	6,54	-2,29	-3,58 a -0,97		Decrescente	
Preta	9,74	8,90	8,83	8,37	8,30	7,93	7,37	7,24	7,77	7,75	6,85	7,34	7,17	7,26	7,43	-1,95	-2,78 a -1,10		Decrescente	
Idade (anos)																				
05 a 06	8,31	8,35	8,41	7,80	7,75	7,53	7,10	6,76	7,07	6,75	6,02	6,97	7,03	6,90	6,94	-1,57	-2,51 a -0,63		Decrescente	
07 a 09	7,40	7,15	7,40	7,33	6,68	7,07	6,45	5,88	6,09	5,79	5,29	5,95	5,70	5,73	5,71	-2,23	-2,92 a -1,54		Decrescente	
05 a 09	8,05	8,25	8,34	7,76	7,52	7,41	6,84	6,36	6,67	6,33	5,68	6,48	6,43	6,37	6,33	-2,12	-3,08 a -1,15		Decrescente	
	Prevalência anual de excesso de peso (%)																			
Sexo																				
Feminino	21,59	22,04	22,91	24,34	24,57	25,07	27,04	27,94	28,52	28,33	28,64	27,37	30,58	32,12	30,19	2,61	2,06 a 3,16		Crescente	
Masculino	25,14	25,63	26,38	27,88	27,82	28,13	29,47	29,62	30,30	30,06	30,65	29,00	32,40	35,11	32,75	1,94	1,52 a 2,35		Crescente	
Raça/cor																				
Amarela	22,43	21,48	22,92	25,93	25,03	26,72	28,10	28,25	28,64	28,45	28,93	28,57	33,15	34,78	32,17	2,96	2,34 a 3,59		Crescente	
Branca	26,66	26,98	27,38	28,95	29,43	29,57	31,14	31,81	31,96	31,73	31,95	30,55	33,85	35,86	33,39	1,78	1,35 a 2,22		Crescente	
Indígena	21,49	21,87	22,18	21,99	22,93	24,84	24,74	26,24	25,76	26,92	26,42	25,97	25,35	31,21	28,55	2,20	1,71 a 2,70		Crescente	
Parda	23,65	23,81	24,22	25,47	25,52	25,81	27,13	28,18	28,90	28,44	28,70	27,27	30,47	32,72	30,77	2,08	1,66 a 2,50		Crescente	
Preta	21,38	21,80	21,17	23,37	22,60	23,50	24,60	24,81	25,44	25,45	26,03	24,46	28,64	30,38	28,66	2,30	1,86 a 2,74		Crescente	
Idade (anos)																				
05 a 06	23,67	24,21	24,97	26,44	26,40	26,75	28,21	28,54	28,85	28,79	29,18	27,20	30,24	32,39	29,89	1,77	1,31 a 2,23		Crescente	
07 a 09	22,72	20,24	20,54	22,19	25,41	26,15	27,75	28,67	29,66	29,25	29,73	28,97	32,72	34,65	32,84	3,43	2,47 a 4,41		Crescente	
05 a 09	23,41	23,88	24,67	26,15	26,19	26,58	28,03	28,60	29,18	28,99	29,43	28,05	31,34	33,41	31,36	2,20	1,79 a 2,62		Crescente	
	Prevalência anual de déficit de altura (%)																			
Sexo																				
Feminino	13,24	12,90	11,91	11,74	10,40	9,38	9,48	8,74	9,37	8,86	8,35	7,58	8,51	8,29	7,54	-3,82	-4,79 a -2,85		Decrescente	
Masculino	15,39	14,84	13,75	13,50	11,93	11,00	10,39	9,36	10,14	9,52	8,95	7,97	9,26	9,11	7,92	-4,52	-5,62 a -3,39		Decrescente	
Raça/cor																				
Amarela	13,13	14,60	14,71	13,15	11,45	10,51	10,21	9,20	9,92	9,25	8,67	6,44	8,85	7,78	6,38	-5,35	-6,30 a -4,40		Decrescente	
Branca	12,34	12,72	11,53	11,29	10,03	9,26	9,10	8,43	9,14	8,80	8,34	7,54	8,41	8,35	7,32	-3,55	-4,53 a -2,55		Decrescente	
Indígena	14,78	17,53	15,48	14,98	12,24	12,08	12,02	12,06	12,58	15,80	12,33	10,91	15,02	15,03	14,05	-0,68	-2,70 a 1,39		Estabilidade	
Parda	14,16	14,23	13,12	12,83	11,30	10,18	9,85	8,97	9,58	9,03	8,58	8,06	8,93	8,81	8,01	-4,05	-5,35 a -2,74		Decrescente	
Preta	14,30	14,71	13,00	13,21	11,74	11,18	9,97	9,22	9,78	9,53	8,83	7,29	9,33	8,30	7,64	-4,55	-5,42 a -3,68		Decrescente	
Idade (anos)																				
05 a 06	13,81	13,94	12,82	12,58	10,97	9,81	9,34	8,69	9,60	8,96	8,58	7,93	8,75	8,77	8,08	-3,84	-5,29 a -2,37		Decrescente	
07 a 09	15,71	13,44	13,14	13,49	11,87	11,15	10,64	9,34	9,74	9,31	8,59	7,55	8,90	8,49	7,35	-4,81	-5,58 a -4,03		Decrescente	
05 a 09	14,34	13,90	12,84	12,64	11,16	10,18	9,85	8,98	9,66	9,11	8,58	7,75	8,82	8,65	7,71	-4,25	-5,33 a -3,15		Decrescente	
	Prevalência anual de desnutrição (%)																			
Sexo																				
Feminino	10,47	10,43	9,98	9,62	8,81	8,24	8,03	7,39	7,86	7,42	6,83	6,87	7,30	7,23	6,83	-3,11	-4,11 a -2,10		Decrescente	
Masculino	11,89	11,69	11,18	10,75	9,87	9,36	8,81	8,11	8,68	8,21	7,60	7,45	8,09	7,87	7,25	-3,49	-4,43 a -2,55		Decrescente	
Raça/cor																				
Amarela	10,73	11,66	11,37	11,19	9,63	8,97	8,67	7,90	8,47	7,88	7,24	6,10	7,39	6,79	6,00	-4,56	-5,31 a -3,79		Decrescente	
Branca	10,11	10,17	9,60	9,14	8,40	7,92	7,58	7,13	7,70	7,37	6,83	6,71	7,17	7,08	6,59	-3,02	-4,03 a -2,01		Decrescente	
Indígena	11,68	13,44	11,09	11,18	9,10	9,83	8,79	8,74	9,04	10,98	8,78	8,54	10,87	10,90	10,19	-1,12	-3,05 a 0,85		Estabilidade	
Parda	11,56	11,39	10,75	10,34	9,39	8,85	8,29	7,59	8,10	7,70	7,23	7,42	7,78	7,69	7,28	-3,30	-4,63 a -1,95		Decrescente	
Preta	12,02	11,81	10,99	11,00	10,02	9,56	8,67	8,23	8,77	8,64	7,84	7,31	8,25	7,78	7,54	-3,39	-4,29 a -2,48		Decrescente	
Idade (anos)																				
05 a 06	11,06	11,15	10,62	10,19	9,36	8,67	8,22	7,73	8,34	7,85	7,30	7,45	7,89	7,84	7,51	-2,83	-4,10 a -1,55		Decrescente	
07 a 09	11,55	10,29	10,26	10,40	9,27	9,11	8,54	7,61	7,92	7,55	6,94	6,75	7,30	7,11	6,53	-3,86	-4,58 a -3,14		Decrescente	
05 a 09	11,19	11,08	10,59	10,20	9,34	8,79	8,35	7,67	8,16	7,72	7,13	7,11	7,63	7,51	7,02	-3,36	-4,40 a -2,30		Decrescente	
	Prevalência anual de dupla carga de má nutrição (desnutrição + excesso de peso) (%)																			
Sexo																				
Feminino	21,26	21,45	21,44	21,8	21,1	20,78	21,55	21,36	22,12	21,59	21,15	20,56	22,58	23,29	21,92	0,30	-0,09 a 0,69		Estabilidade	
Masculino	24,46	24,51	24,38	24,7	23,78	23,43	23,55	22,93	23,83	23,24	22,93	21,95	24,29	25,43	23,63	-0,23	-0,76 a 0,30		Estabilidade	
Raça/cor																				
Amarela	21,95	22,43	22,84	24,16	22,15	22,33	22,72	22,02	22,79	22,11	21,7	20,39	23,97	24,18	22,08	-0,01	-0,56 a 0,54		Estabilidade	
Branca	23,45	23,67	23,3	23,63	23,13	22,71	23,15	23,03	23,68	23,24	22,8	21,98	24,09	25,01	23,28	0,06	-0,33 a 0,45		Estabilidade	
Indígena	22,44	24,38	22,18	22,18	20,56	22,25	21,16	21,86	21,92	24,43	21,98	21,53	23,55	26,5	24,47	0,61	-0,28 a 1,52		Estabilidade	
Parda	23,39	23,3	22,86	23,08	22,15	21,76	21,86	21,68	22,56	21,92	21,58	21,05	23,02	24,05	22,66	-0,16	-0,78 a 0,46		Estabilidade	
Preta	22,71	22,71	21,58	22,69	21,33	21,3	20,97	20,64	21,49	21,37	20,85	19,55	22,57	22,97	21,87	-0,22	-0,85 a 0,41		Estabilidade	
Idade (anos)																				
05 a 06	22,89	23,26	23,1	23,41	22,56	22,05	22,32	22,00	22,76	22,25	21,89	21,05	23,01	24,03	22,45	-0,15	-0,62 a 0,32		Estabilidade	
07 a 09	22,91	20,42	20,57	21,53	21,99	22,19	22,42	21,94	22,75	22,18	21,81	21,23	23,66	24,44	22,95	0,61	0,12 a 1,10		Crescente	
05 a 09	22,90	23,02	22,93	23,28	22,44	22,08	22,36	21,97	22,75	22,22	21,85	21,14	23,3	24,22	22,70	-0,04	-0,54 a 0,45		Estabilidade	

APC: variação percentual anual; IC95%: intervalo de confiança de 95%.

Fonte: dados do Sisvan (2008–2022).

As prevalências de excesso de peso apresentaram tendência crescente em todas as categorias analisadas. Quanto à prevalência de DCMN, a faixa de 7 a 9 anos foi a única categoria a ter tendência de crescimento da DCMN. Apesar da tendência de estabilidade nas demais categorias investigadas, destacam-se as altas prevalências da DCMN no SB ao longo do período analisado (Tabela 2).

Na Figura 2, essas altas prevalências da DCMN no SB ficam ainda mais evidentes. Na evolução temporal avaliada, as prevalências da DCMN estão próximas às de excesso de peso, enquanto as prevalências de emaciação, déficit de altura e desnutrição estão em queda.

**Figura 2:** Evolução temporal da prevalência de excesso de peso, emaciação, déficit de altura, desnutrição e dupla carga de má nutrição em crianças de 5 a 9 anos na região do semiárido brasileiro, Brasil, 2008–2022.

DCMN: dupla carga de má nutrição.

Fonte: dados do Sisvan (2008–2022).

Quanto à distribuição espacial, houve um aumento na prevalência do excesso de peso na região SB entre os anos de 2008 e 2022, de 23,41% para 31,36%, com destaque para as Unidades Federativas mais ao norte da região, como Ceará e Rio Grande do Norte, onde as cores mais escuras no mapa indicam prevalências mais altas. Em relação à desnutrição, houve uma redução nas prevalências em toda a região SB, com valores de 11,19% em 2008 e 7,02% em 2022. No caso da DCMN, a prevalência apresentou uma queda entre 2008 (22,90%) e 2015 (21,97%), seguida de um aumento entre 2015 e 2022 (22,70%), retornando a níveis semelhantes aos observados em 2008 (Figura 3).

**Figura 3:** Evolução temporal da prevalência (%) do excesso de peso (A,B,C), desnutrição (D,E,F) e dupla carga de má nutrição (G,H,I) em crianças de 5 a 9 anos no semiárido brasileiro, de acordo com as Unidades Federativas, Brasil, 2008, 2015, 2022.

Fonte: dados do Sisvan (2008, 2015, 2022).

## DISCUSSÃO

Os resultados do presente estudo mostram elevada prevalência da DCMN no SB (22,70%), e também no NSB (21,47%), em 2022. A evolução temporal desta prevalência no SB foi crescente para crianças de 7 a 9 anos e estável para menores de 7 anos, acompanhando as prevalências do excesso de peso, que seguiu tendência crescente em todas as regiões, sexo, faixas etárias e raça/cor. Por outro lado, a desnutrição apresentou tendência decrescente de estabilidade em todas as regiões, com exceção da população indígena do SB. A DCMN é um fenômeno complexo, que está intimamente ligado a baixos níveis socioeconômicos e à acelerada transição nutricional no SB. Essa transição pode ser explicada, em parte, pela redução do consumo de alimentos *in natura*, sendo substituídos pelos mais processados, ricos em gorduras e açúcares simples na população infantil^22^.

A tendência crescente da DCMN em crianças de 7 a 9 anos, faixa etária que demonstrou maior APC na prevalência de excesso de peso, é preocupante. Nesse cenário, os indivíduos e a sociedade sofrem as consequências tanto da desnutrição quanto do excesso de peso^1^. De Sanctis et al.^23^ demonstraram que a desnutrição na infância, causada por baixa ingestão calórica e fatores genéticos, altera o balanço energético, a oxidação e o acúmulo de gordura em fases futuras, o que aumenta a predisposição à obesidade. Wells et al.^1^ corroboram esses achados, relacionando a desnutrição infantil a alterações na microbiota intestinal e em órgãos como fígado e rins, além de predispor a criança ao excesso de peso^1,23^. Assim como a desnutrição, o excesso de peso afeta o metabolismo da insulina e o controle da saciedade, devido à ação da leptina no hipotálamo, moldando as preferências alimentares e a microbiota intestinal^1,23^. Tal quadro eleva o risco de desenvolvimento de DCNT e impacta a saúde de gerações futuras, uma vez que ambas as condições possuem efeitos intergeracionais^1,23^. Esses achados reforçam a necessidade de considerar a desnutrição e o excesso de peso como condições coexistentes, originadas de fatores comuns e com consequências interligadas^1^.

As APC do estado nutricional das crianças na região do SB foram mais acentuadas ao longo do período analisado tanto para o excesso de peso (APC = 2,20%; IC95% 1,79 a 2,62) quanto para a desnutrição (APC = -3,36%; IC95% -4,40 a -2,30). Santos e Fontão^2^ destacam a relação da transição nutricional no Brasil com a mudança nos padrões alimentares, observando um aumento médio de 5,5% no consumo de alimentos ultraprocessados em um período de 10 anos (2008–2018), enfatizando que essa mudança é mais acentuada em regiões de maior vulnerabilidade socioeconômica, onde o acesso a alimentos saudáveis é limitado. Popkin et al.^3^, por sua vez, apontam que a DCMN tem aumentado em mais de 1 ponto percentual por ano nos países de baixa e média renda, devido ao crescimento do excesso de peso. A desigualdade social encontrada na população do SB é evidenciada ao analisar a distribuição do PIB *per capita*. Um estudo de 2017 mostrou que cerca de pouco mais de 200 municípios pertencentes à região do SB, na época, apresentaram um valor superior a um salário mínimo^18^. Esses fatores estão diretamente vinculados à prevalência de insegurança alimentar e deficiências nutricionais, já que afetam consideravelmente o acesso a alimentos saudáveis por parte da população^18^_._ Dessa forma, esses resultados servem como alerta para a necessidade de ações mais efetivas direcionadas para a região do SB, que carece de maior assistência, visto que o excesso de peso e a desnutrição compartilham determinantes e condicionantes comuns no processo saúde-doença^22^.

Outro ponto de alerta levantado pelos resultados do presente estudo são as diferenças raciais encontradas para cada condição. Crianças indígenas apresentaram uma menor APC nas prevalências de emaciação, déficit de altura e desnutrição, com tendência de estabilidade. Em contraste, houve uma tendência crescente da prevalência de excesso de peso, reforçando a transição nutricional e a desassistência nesse grupo, com a perpetuação da má nutrição em todas as suas formas. Sabe-se que a população indígena é uma das mais afetadas pela desigualdade social no Brasil, influenciada por fatores como luta territorial, dificuldade de acesso aos serviços de saúde – causada pela distância das unidades básicas de saúde e busca tardia por assistência –, baixa renda, além de fatores culturais, o que dificulta o monitoramento de seu estado nutricional^24^.

A acelerada transição nutricional com a sobreposição de problemas de saúde pública e a coexistência de desvios nutricionais em uma mesma população são preocupações desde a chamada nutricional que ocorreu no Brasil há cerca de 20 anos^25^. Atualmente, essa apreensão se confirma nos mapas e gráfico deste estudo, nos quais é possível observar a influência da redução da desnutrição (que passou de 11,19% em 2008 para 7,02% em 2022 no SB) e o aumento do excesso de peso (que subiu de 23,41% em 2008 para 31,36% em 2022 no SB) na evolução temporal da DCMN. Isso evidencia a rápida transição nutricional enfrentada pelo SB, no período analisado, notando-se uma velocidade de aumento do excesso de peso duas vezes maior do que a velocidade de queda da desnutrição em crianças da região. Outro ponto que reforça como essa transição está ocorrendo de forma moderadamente mais rápida no SB em comparação com o NSB é a prevalência de déficit de altura em crianças, com APC de -4,25% (IC95% -5,33 a -3,15) no SB, e APC de -3,85% (IC95% -4,63 a -3,06) na região NSB. Ademais, o crescimento da prevalência de excesso de peso também foi mais acelerado no SB, com APC de 2,20% (IC95% 1,79 a 2,62), demonstrando que a região, que comumente enfrentava a desnutrição, agora lida com o aumento acelerado de sobrepeso e obesidade. De forma semelhante, Rocha^26^ (2016), utilizando dados de inquéritos de corte transversal em série e de base populacional do SB de 1987 a 2007, demonstrou que a desnutrição aguda diminuiu drasticamente: de 12,4% (IC95% 11,4 a 13,4) para 4,3% (IC95% 3,3 a 5,4), enquanto a obesidade infantil aumentou de forma alarmante, quase 240%, com tendência temporal de aumento de 12,01%. Silva^27^ (2019) realizou uma análise longitudinal dos municípios brasileiros beneficiários do Programa Bolsa Família e também demonstrou o estágio avançado da transição nutricional brasileira, caracterizado pelo aumento médio de 17,2% para 18,4% no excesso de peso, tanto isoladamente quanto coexistindo com a desnutrição (ex. DCMN), principalmente entre as crianças das camadas socioeconomicamente mais vulneráveis da população. Ainda, segundo o estudo, a prevalência de DCMN foi associada a fatores socioeconômicos, como PIB *per capita*, expectativa de anos de estudo, taxa de desemprego e aglomeração domiciliar^27^.

Apesar do cenário complexo de desigualdade socioeconômica no Brasil, as políticas sociais inclusivas, implementadas a partir de 2003, como o programa Bolsa Família, contribuíram significativamente para a redução da insegurança alimentar e promoveram mudanças nos padrões alimentares da população^14,24^. A melhora nas condições socioeconômicas pode ter gerado uma melhoria na nutrição e consequentemente na altura das crianças avaliadas, mas, ao mesmo tempo, essas mudanças podem estar relacionadas a um aumento na disponibilidade de alimentos calóricos e ultraprocessados, e consequentemente ao aumento do excesso de peso, como mostram os resultados deste estudo. Embora tenham ocorrido significativas mudanças socioeconômicas, a crise política e econômica enfrentada recentemente gerou um impacto expressivo na situação alimentar e nutricional do país, especialmente entre indivíduos mais vulneráveis, evidenciado pelo aumento do desemprego, inflação e insegurança alimentar – fatores que influenciam na DCMN^26,28,29^. Diante desse cenário, para que as ações de profissionais e gestores de saúde sejam eficazes, os problemas socioeconômicos, culturais, políticos e de saúde devem ser compreendidos de forma interligada^28^. Esses achados ressaltam a importância de políticas públicas que visem reduzir a desigualdade social e melhorar o estado nutricional das crianças mais vulneráveis.

Este estudo teve limitações inerentes à utilização de dados secundários, como a qualidade insuficiente e a baixa cobertura do Sisvan em algumas regiões^11,30^. Um trabalho recente que avaliou a evolução da cobertura no Sisvan entre 2008 e 2021 encontrou o seguinte: desde que a plataforma Sisvan-Web foi implementada em 2008, ele aumentou sua cobertura para todas as regiões e faixas etárias até 2019^11,30^. No entanto, em 2020, primeiro ano da pandemia de covid-19, a cobertura diminuiu substancialmente, com recuperação em 2021 para as faixas de 0 a 4 e de 5 a 9 anos^11,30^.

Apesar dessas limitações que podem influenciar nossos resultados, este estudo reforça a importância do acompanhamento do estado nutricional e a atitude de vigilância desde a infância, além de políticas e programas governamentais que atuem de fato na população mais vulnerável, prevenindo assim novos agravos e tratando dos já existentes, bem como contribuindo para a erradicação da fome e o aumento da qualidade de vida de cada indivíduo, especialmente para os mais vulneráveis. Ademais, este estudo destaca-se pelo ineditismo, visto que são escassas as pesquisas que tratam da magnitude da coexistência dos desvios nutricionais (desnutrição e excesso de peso) no território. Além disso, embora tenhamos avançado no estudo da desnutrição, avaliando os índices relacionados ao peso e altura, não conseguimos avaliar todas as suas formas existentes, como carências de micronutrientes. Sendo assim, mais estudos são necessários para investigar a desnutrição em todo seu escopo.

Em conclusão, a DCMN, com a presença simultânea de desnutrição e excesso de peso, é uma problemática ainda mais desafiadora em um país continental como o Brasil e principalmente na região do SB, com baixos níveis socioeconômicos e, ao mesmo tempo, em um estágio de transição nutricional acelerado. Apesar dos avanços na saúde pública, é preciso priorizar a Agenda de Alimentação e Nutrição, com enfoque desde o início do ciclo da vida, intervenções direcionadas à promoção da saúde e prevenção da má nutrição em todas as suas formas, especialmente na infância e em populações vulneráveis, como as crianças indígenas.

## Data Availability

Dados brutos do artigo estão disponíveis mediante solicitação ao autor correspondente.

## Footnotes

a Disponível em: https://sisaps.saude.gov.br/sisvan/relatoriopublico/
